# Absolute Contusion Expansion Is Superior to Relative Expansion in Predicting Traumatic Brain Injury Outcomes: A Multi-Center Observational Cohort Study

**DOI:** 10.1089/neu.2023.0274

**Published:** 2024-02-27

**Authors:** Alexander Fletcher-Sandersjöö, Teodor Svedung Wettervik, Charles Tatter, Jonathan Tjerkaski, David W. Nelson, Marc Maegele, Mikael Svensson, Anders Lewén, Per Enblad, Bo-Michael Bellander, Eric Peter Thelin

**Affiliations:** ^1^ Department of Neurosurgery, Karolinska University Hospital, Stockholm, Sweden.; ^2^ Department of Clinical Neuroscience, Section of Perioperative Medicine and Intensive Care, Karolinska Institutet, Stockholm, Sweden.; ^3^ Department of Medical Sciences, Section of Neurosurgery, Uppsala University, Uppsala, Sweden.; ^4^ Department of Radiology, Stockholm Southern Hospital, Stockholm, Sweden.; ^5^ Function Perioperative Care and Medicine, Karolinska University Hospital, Stockholm, Sweden.; ^6^ Department of Physiology and Pharmacology, Section of Perioperative Medicine and Intensive Care, Karolinska Institutet, Stockholm, Sweden.; ^7^ Department for Trauma and Orthopedic Surgery, Cologne-Merheim Medical Center, University Witten/Herdecke, Cologne, Germany.; ^8^ Institute for Research in Operative Medicine, University Witten/Herdecke, Cologne, Germany.; ^9^ Department of Neurology, Karolinska University Hospital, Stockholm, Sweden.

**Keywords:** contusion, contusion expansion, hematoma expansion, lesion progression, outcome, traumatic brain injury

## Abstract

Contusion expansion (CE) is a potentially treatable outcome predictor in traumatic brain injury (TBI), and a suitable end-point for hemostatic therapy trials. However, there is no consensus on the definition of clinically relevant CE, both in terms of measurement criteria (absolute vs. relative volume increase) and cutoff values. In light of this, the aim of this study was to assess the predictive abilities of different CE definitions on outcome. We performed a multi-center observational cohort study of adults with moderate-to-severe TBI treated in an intensive care unit. The exposure of interest was CE, defined as the absolute and relative volume change between the first and second computed tomography scan. The primary outcome was the Glasgow Outcome Scale (GOS) at 6–12 months post-injury, dichotomized into unfavorable (GOS ≤3) or favorable (GOS ≥4). The secondary outcome was all-cause mortality. In total, 798 patients were included, with a median duration of 7.0 h between the first and second CT scan. The median absolute and relative CE was 1.5 mL (interquartile range [IQR] 0.1–8.3 mL) and 100% (IQR 10–530%), respectively. Both CE forms were independently associated with unfavorable GOS. Absolute CE outperformed relative CE in predicting both unfavorable GOS (area under the curve [AUC]: 0.65 vs. 0.60, *p* = 0.002) and all-cause mortality (AUC: 0.66 vs. 0.60, *p* = 0.003). For dichotomized CE, absolute cutoffs of 1–10 mL yielded the best results. We conclude that absolute CE demonstrates stronger outcome correlation than relative CE. In studies focusing on lesion progression in TBI, it may be advantageous to use absolute CE as the primary outcome metric. For dichotomized outcomes, cutoffs between 1 and 10 mL are suggested, depending on the desired sensitivity-specificity balance.

## Introduction

Contusion expansion (CE) is a potentially modifiable outcome predictor in traumatic brain injury (TBI),^1^ making it an important surrogate end-point for hemostatic trials that are underpowered for patient-centric outcomes. Despite this, the research community is divided on the definition of clinically relevant CE,^2^ which impacts interpretation and comparison of study results. Different approaches have been employed, with some using absolute contusion expansion in milliliters (mL),^3,4^ others adopting relative expansion in percentage,^5–9^ and a few combining both methods.^10,11^ Although research on spontaneous intracerebral hemorrhage (ICH) suggests that absolute volume change has a stronger association to outcome than relative change,^12^ it is uncertain whether this can be extrapolated to traumatic contusions. Identifying the most appropriate CE definition and cutoffs would clarify the clinical relevance of hematoma reduction and foster consistency in trial designs.

In light of the foregoing, this study aimed to evaluate the predictive abilities of various CE definitions on patient outcomes in TBI. Drawing from research on spontaneous ICH, we hypothesized that absolute CE would outperform relative CE.

## Methods

### Study design and setting

This retrospective multi-center observational cohort study included adults (≥15 years) with moderate-to-severe TBI treated in an intensive care unit at two neurosurgical centers in Sweden between 2006 and 2021 (Center 1) or between 2008 and 2018 (Center 2). Moderate-to-severe TBI was defined as a Glasgow Coma Scale^13^ (GCS) score of 3–13 on hospital admission. The study hospitals are the only neurosurgical centers in their regions and serve a combined population of 4,500,000 (43% of the Swedish population). Patients were excluded if they did not show any contusions, follow-up data were missing, primary treatment was initiated at another hospital, injury was penetrating, the first computed tomography (CT) scan was conducted >12 h post-injury, there was absence of a second CT scan, or if they underwent contusion evacuation before a second CT scan was performed. Both centers generally adhere to the guidelines established by the Brain Trauma Foundation,^14^ where unconscious patients are mechanically ventilated and intracranial pressure is monitored.

The study was approved by the Swedish Ethical Review Authority (Center 1 Dnr: 2019-04476, Center 2 Dnr: 2022-06526-02). Informed consent was not required for Center 1. In Center 2, written informed consent was obtained from most patients or their relatives during treatment or follow-up, but the requirement was waived if the patient or relatives could no longer be contacted.

### Outcome and exposure

The primary outcome was the Glasgow Outcome Scale (GOS),^15^ assessed at a median of 8.0 months (interquartile range [IQR] 6.0–12) after injury, and subsequently dichotomized into unfavorable (GOS score ≤3, representing severe disability, vegetative state, or death) or favorable (GOS score ≥4, indicating moderate disability or good recovery). The secondary outcome was all-cause mortality, evaluated at the same time point as the primary outcome.

The exposure of interest was CE, defined as the absolute or relative increase in contusion volume between the first and second CT scan. The second CT scan was normally conducted 6–8 h after the initial CT scan or earlier if clinically indicated. For patients who did not have a contusion on their initial CT scan (*n* = 77), only absolute CE could be calculated. If a patient was initially admitted to another hospital and then transferred to one of our centers, the CT scan performed at the first hospital was analyzed. If multiple contusions were present, their volumes were combined.

### Data collection and contusion volume calculation

Patients were identified through local databases that included all those admitted to each department with a TBI. Clinical data were obtained from electronic medical records software, and imaging data were retrieved from the radiological management software Sectra Picture Archiving and Communication System (PACS) IDS7 (Sectra AB, Linköping, Sweden). Dedicated research nurses prospectively collected GOS data via validated questionnaires through mail and telephone interviews. Contusion volumes were calculated using semi-automated threshold-guided planimetry, excluding the surrounding edema. In Center 1, this was performed using the IDS7 software, as previously described,^1^ while the BrainLab^®^ elements software^16^ was used in Center 2 (BrainLab Germany Headquarters, Munich, Germany). Each center had one assessor who was blinded to patient outcomes, but was made aware of whether the CT scan they were analyzing was the initial or follow-up one. The latter was considered essential to ensure consistent definition of hematoma boundaries across scans.

### Statistical analysis

As all continuous data deviated from a normal distribution pattern (Shapiro–Wilks test *p* value <0.05), we present them as median (IQR), and categorical data are presented as counts (percentages).

To assess the agreement between the IDS7 and BrainLab^®^ elements methods for measuring contusion volume, we calculated intraclass correlation coefficients (ICC) for 30 randomly selected patients from Center 1, in whom volumes were determined by a single assessor using both methods. We employed a two-way mixed effects model based on single measurements and absolute agreement for the ICC calculation,^17^ using the R-package “irr”.^18^

Multivariable logistical regression analyses were performed to determine the association between CE and outcome, adjusting for baseline contusion volume and variables from the core and CT International Mission for Prognosis and Clinical Trials in Traumatic Brain Injury (IMPACT) model (age, GCS, pupillary status, Marshall CT classification, subarachnoid hemorrhage, and epidural hemorrhage^19^). Listwise deletion was used to handle missing variables.

To compare the predictive value of absolute and relative CE for unfavorable GOS and all-cause mortality, we generated receiver operating characteristic (ROC) curves and compared their discriminatory performance using the DeLong method.^20^ Sensitivity, specificity, and Youden's index for several commonly used cutoff definitions of CE were also calculated. In a sensitivity analysis we then conducted subgroup evaluations in patients with baseline contusion volumes ≥1 mL, severe TBI (GCS ≤8), and for each center separately. A univariable logistical regression model with interaction-term analyses was also used to investigate potential effect modifiers. Isolated TBI was defined as a moderate-to-severe TBI with an extracranial Abbreviated Injury Scale (AIS)^21^ ≤ 2.

All statistical analyses were conducted using R software version 4.1.2, and *p* < 0.05 was considered statistically significant.

## Results

### Baseline data

**FIG. 1.**
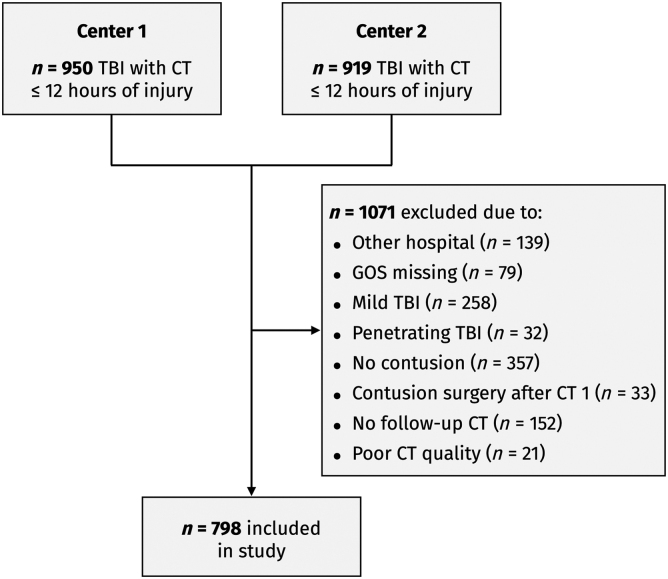
Patient inclusion flow-chart. CT, computed tomography; GOS, Glasgow Outcome Scale; TBI, traumatic brain injury

A total of 1869 patients were screened, out of whom 798 were included (Fig. 1). The median age was 52 years, and 78% were male. There was excellent agreement between the IDS7 and BrainLab^®^ methods for contusion volume calculations, with an ICC of 0.99 (0.97–0.99). Table S1 provides a detailed overview of the antithrombotic agents used and their hemostatic optimization. The median baseline contusion volume was 0.7 ml (IQR 0.1–3.0 mL), and the median absolute and relative CE were 1.5 mL (IQR 0.1–8.3 mL) and 100% (IQR 10–530%), respectively. At follow-up, 44% exhibited unfavorable GOS, and mortality was 19%. No major differences were observed in the data from the two included centers, with the exception that Center 2 had a larger baseline contusion volume and absolute CE (Table 1).

**Table 1.** Characteristics of the Study Cohort

Variable	All patients (*n* = 798)	Center 1 (*n* = 454)	Center 2 (*n* = 344)
Baseline data			
Age (years)	52 (30–62)	47 (28–62)	52 (37–67)
Male sex	619 (78%)	347 (76%)	272 (79%)
Anticoagulant treatment	33 (4.2%) (5 missing)	15 (3.3%) (5 missing)	18 (5.2%)
Antiplatelet treatment	60 (7.6%) (5 missing)	32 (7.1%) (5 missing)	28 (8.1%)
Glasgow Coma Scale (GCS)	7 (5–10)	6 (3–9)	7 (7–10)
Moderate TBI (GCS 3–8)	252 (32%)	128 (28%)	124 (36%)
Severe TBI (GCS 9–12)	546 (68%)	326 (72%)	220 (64%)
Bilateral non-reactive pupils	58 (7.3%)	38 (8.4%)	20 (5.8%)
Treatment			
Invasive neuromonitoring	599 (75%)	331 (73%)	268 (78%)
Craniotomy	323 (40%)	175 (39%)	148 (43%)
Decompressive craniectomy	72 (9.0%)	37 (8.1%)	35 (10%)
Radiology			
Isolated TBI	309 (68%) (344 missing)	309 (68%)	Data not collected
Marshall CT classification			
Class II	417 (52%)	214 (47%)	203 (59%)
Class III	64 (8.0%)	43 (9.5%)	21 (6.1%)
Class IV-VI	317 (40%)	197 (43%)	120 (35%)
First CT contusion volume (mL)	0.7 (0.1–3.0)	0.5 (0.1–1.6)	1.3 (0.1–7.3)
≥ 1 mL	351 (44%)	167 (37%)	184 (53%)
Second CT contusion volume (mL)	3.7 (0.6–15)	2.0 (0.4–8.0)	6.6 (1.5–27)
Hours between first and second CT	7.0 (5.0–11)	6.4 (4.5–9.3)	8.0 (6.0–12.0)
Absolute CE (mL)	1.5 (0.1–8.3)	0.7 (0.0–5.7)	3.3 (0.4–15)
Relative CE (%)	100 (0–530)	100 (0–650)	90 (10–420)
Outcome			
Months to outcome assessment	8.0 (6.0–12)	12 (5.0–13)	7.0 (6.0–8.0)
Unfavorable GOS	354 (44%)	195 (43%)	159 (46%)
GOS 1 (death)	148 (19%)	87 (19%)	61 (18%)
GOS 2	7 (0.9%)	4 (0.9%)	3 (0.9%)
GOS 3	199 (25%)	104 (23%)	95 (28%)
GOS 4	248 (31%)	167 (37%)	81 (24%)
GOS 5	196 (25%)	92 (20%)	104 (30%)

Data presented as median (interquartile range) or number (proportion). Isolated TBI defined as extracranial Abbreviated Injury Scale ^21^ ≤ 2.

CE, contusion expansion; CT, computed tomography; GOS, Glasgow Outcome Scale; TBI, traumatic brain injury.

### Relationship between CE and outcome

Both absolute and relative CE showed independent association with unfavorable GOS after adjusting for baseline contusion volume and the core and CT IMPACT model. Pseudo-*R^2^* was 0.068 and 0.032 for absolute and relative CE, but only 0.008 for baseline contusion volume, highlighting the clinical importance of CE as compared with initial contusion volume. When all-cause mortality was the outcome, a significant association was only seen for absolute CE (Table 2). Tables S2 and S3 show pseudo-*R^2^* as well as uni- and multivariable odds ratio (OR) and *p* values for all variables included in the regression analysis.

**Table 2.** Multivariable-Adjusted Relationship Between Contusion Expansion (CE) and Unfavorable Glasgow Outcome Scale or All-Cause Mortality

Variable	Unfavorable GOS			All-cause mortality		
	OR (95% CI)	*p *value	pseudo* R^2^*	OR (95% CI)	*p *value	pseudo* R^2^*
Absolute CE (mL)	1.02 (1.01–1.04)	**< 0.001**	0.068	1.01 (1.00–1.02)	**0.024**	0.045
Relative CE (%)	1.01 (1.00–1.02)	**0.008**	0.032	1.01 (1.00–1.01)	0.067	0.021

Bold text in the *p* value column indicates a statistically significant correlation (*p* < 0.05). OR and *p* values presented are those following multivariable adjustment for baseline contusion volume, age, Glasgow Coma Scale on admission, pupil responsiveness, Marshall computed tomography classification, presence of subarachnoid hemorrhage, and presence of epidural hemorrhage.

CI, confidence interval; OR, odds ratio.

**FIG. 2.**
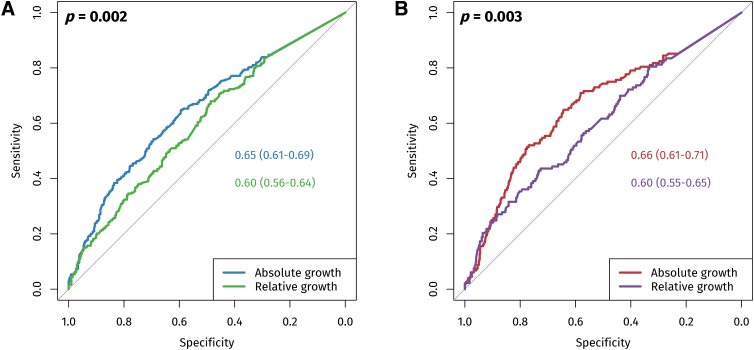
Receiver operating characteristic (ROC) curves for absolute and relative contusion expansion predicting unfavorable Glasgow Outcome Scale (GOS) score **(A)** and all-cause mortality **(B)**.

ROC curves for absolute and relative CE are presented in Figure 2. For unfavorable GOS, the area under the curve (AUC) was 0.65 (0.61–0.69) for absolute CE and 0.60 (0.56–0.64) for relative expansion, with absolute CE significantly better at discriminating outcomes (*p* = 0.002, Fig. 2A). This pattern persisted when all-cause mortality was employed as the outcome measure as well (AUC 0.66 vs. 0.60, *p* = 0.003, Fig. 2B).

### Sensitivity analyses

**FIG. 3.**
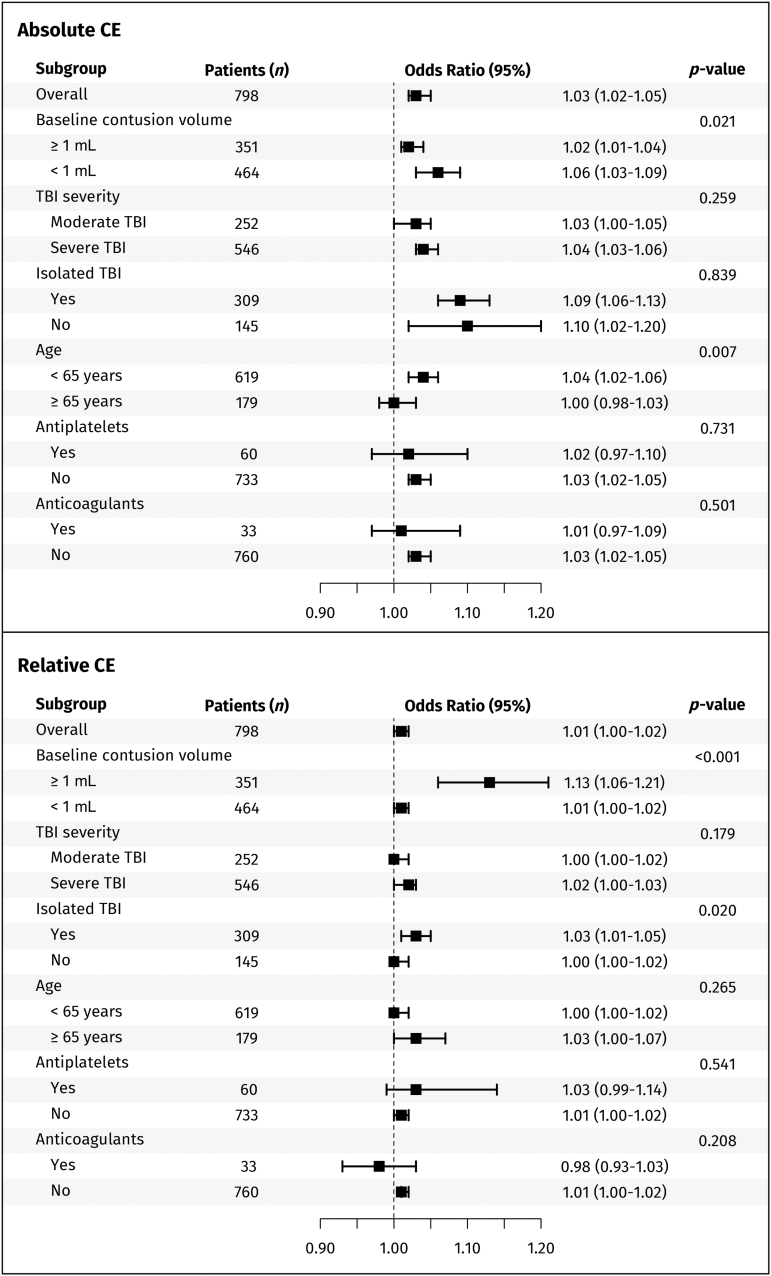
Subgroup analyses and interaction terms for predicting unfavorable Glasgow Outcome Scale (GOS) score using absolute and relative contusion expansion (CE). *P* values denote interaction-term analyses.

Interaction-term analyses showed that absolute CE performed particularly well in patients with initial contusion volumes <1 mL (*p* = 0.021) and for those <65 years of age (*p* = 0.007), whereas TBI severity and extracranial injuries did not influence this effect. In contrast, the performance of relative CE improved in patients with baseline contusion volumes ≥1 mL (*p* < 0.001) and in isolated TBI (*p* = 0.020). The use of pre-injury antiplatelet and anticoagulant therapy did not significantly alter the effects of either absolute or relative CE (Fig. 3).

**FIG. 4.**
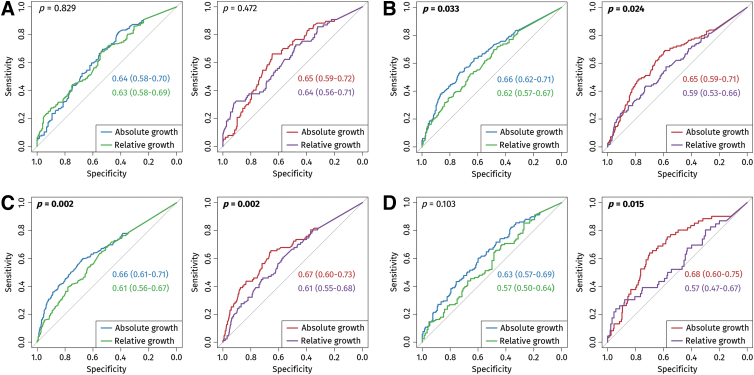
Receiver operating characteristic (ROC) curves for absolute and relative contusion expansion (CE) predicting unfavorable Glasgow Outcome Scale (GOS) score (green-blue) and all-cause mortality (red-purple) in patients with baseline contusion volumes ≥1 **(A)**, severe traumatic brain injury (TBI) **(B)**, Center 1 **(C)**, and Center 2 **(D)**. Bold text marks significant *p* values (< 0.05).

Subgroup analyses revealed that absolute CE continued to outperform relative CE in patients with severe TBI and across each center separately, except for the prediction of unfavorable GOS in Center 2 where the comparison was non-significant. However, in line with the interaction-term analysis, there was no significant difference in their performance when patients with baseline contusion volumes <1 mL were excluded (Fig. 4).

### Optimal CE cutoffs

We calculated the sensitivity, specificity, and Youden's index for various absolute CE cutoffs. As the cutoff increased, specificity rose while sensitivity decreased. Youden's index remained relatively consistent (± 0.03) for cutoffs between 1.0 and 10 mL, before then decreasing (Table 3).

**Table 3.** Sensitivity and Specificity of Commonly Used Absolute CE Cutoffs for the Prediction of Unfavorable Glasgow Outcome Scale

CE cutoff	*n *(%)	Unfavorable GOS			All-cause mortality		
		Sensitivity	Specificity	Y.I.	Sensitivity	Specificity	Y.I.
≥ 1 mL	433 (54%)	0.67	0.56	0.22	0.74	0.50	0.25
≥ 5 mL	263 (33%)	0.45	0.77	0.22	0.55	0.72	0.27
≥ 10 mL	170 (21%)	0.32	0.87	0.19	0.41	0.83	0.24
≥ 20 mL	88 (11%)	0.17	0.94	0.11	0.21	0.91	0.13
≥ 30 mL	58 (7.3%)	0.12	0.96	0.07	0.15	0.94	0.09

CE, contusion expansion; GOS, Glasgow Outcome Scale; Y.I., Youden's index.

**FIG. 5.**
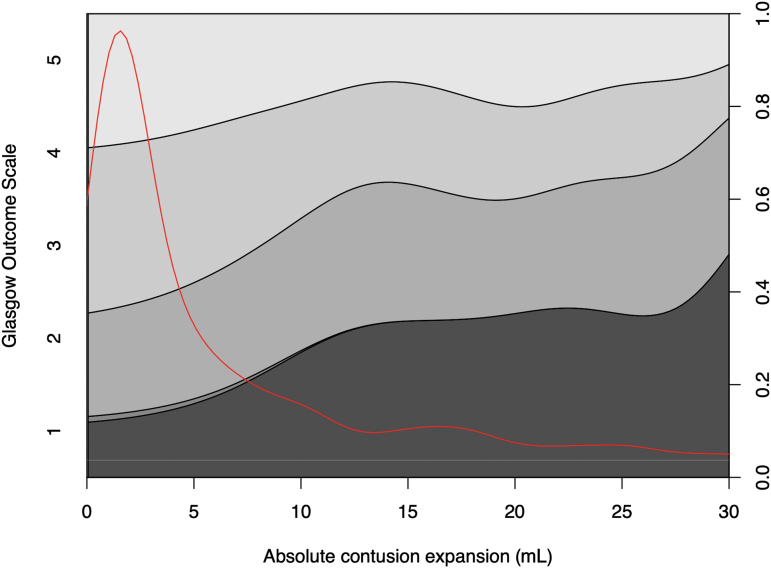
Conditional density plot illustrating the correlation between Glasgow Outcome Score stages (y-axis) and absolute contusion expansion (CE). The red line depicts data distribution.

Figure 5 illustrates the relationship between larger absolute CE (x-axis) and a higher proportion of patients with lower GOS (y-axis). This plot also highlights that even minor absolute CE influenced patient outcomes.

## Discussion

This study aimed to compare the predictive ability of different CE definitions in TBI. The findings support our hypothesis that absolute CE is better than relative CE at predicting unfavorable GOS and all-cause mortality. This aligns with research on spontaneous ICH and should help harmonize the design of future studies.

There is no consensus on how CE should be measured in TBI.^2^ For example, a CRASH-3 trial sub-study used a relative cutoff of 25% to define hematoma expansion,^6^ and Rowell and coworkers' out-of-hospital tranexamic acid trial defined it as a >33% relative increase.^7^ In contrast, the study of recombinant factor VIIA in TBI used continuous absolute CE as the outcome.^3^ Other observational trials aimed at assessing predictors of CE and its impact on outcome have all used different forms of relative or absolute definitions and cutoffs. ^8–11,22–24^ A uniform CE definition would streamline trial designs, prevent for post-hoc analyses, and enhance the likelihood of successful treatment translating to better patient outcomes.

We found that absolute CE was better than relative CE at predicting unfavorable GOS and all-cause mortality. This is consistent with spontaneous ICH findings,^12^ and may be the result of absolute CE's direct correlation with increased intracranial pressure and brain tissue damage. However, both absolute and relative CE performed equally in cases with baseline contusion volumes ≥1 mL, suggesting that the choice between these measures may depend on patient characteristics and study design. Specifically, for contusions in the 0.1–1.0 mL range, absolute CE seems to provide a more precise and consistent measure, making it the favored approach. However, for larger contusions, the efficacy between the two measures evens out. In these cases, researchers and clinicians are afforded the latitude to opt for either absolute or relative CE, contingent on the distinct requirements and goals of their study. However, it is worth noting that although relative CE can be valuable in studies that hinge on certain baseline contusion volumes, such a criterion will limit patient inclusion and study generalizability. For context, requiring a baseline volume of ≥1 mL for inclusion in our study would have omitted 56% of the participants (447 out of 798 patients).

For trials requiring a dichotomized outcome, setting optimal CE cutoffs is vital. An ideal cutoff should have a high specificity —ensuring a high likelihood of poor outcomes in patients exceeding the cutoff—while maintaining a reasonable detection rate. Our analysis revealed a stable Youden's index for cutoffs between 1.0 and 10 mL, beyond which it declined. This suggests that a single optimal cutoff for absolute CE may not exist, and future studies would benefit from utilizing continuous absolute CE instead. If a binary definition is necessary, the selection should fall within the 1.0–10 mL range, bearing in mind the balance between sensitivity and specificity.

Our cohort's median baseline contusion volume was 0.7 mL, similar to the 1.0 mL baseline in the CRASH-3 trial.^6^ We integrated data from two neurosurgical centers, with Center 2 showing higher baseline contusion volumes (1.3 mL vs. 0.5 mL) and absolute CE (3.3 mL vs. 0.7 mL). This disparity likely arises from geographical selection bias. Center 1, in a populated urban area, mandates all moderate-to-severe TBI patients to undergo initial trauma assessment at the hospital. Center 2, in a less populated city, allows initial assessment at smaller, rural hospitals, leading to selective admissions favoring larger contusion volumes. This bias is corroborated by Center 2's higher rates of invasive neuromonitoring (78% vs. 73%), craniotomies (43% vs. 39%), and unfavorable GOS (46% vs. 43%). Additionally, the extended interval between initial and second CT scans at Center 2 (8.0 vs. 6.4 h), likely the result of further referral distances, could have led to larger CE.

### Limitations

We excluded patients who underwent contusion evacuation before the second CT (*n* = 33), who were often younger patients with larger contusions. However, this mirrors the eligibility criteria that would be expected in future hemostatic trials using CE as the primary outcome. Second, follow-up CT scan times varied. However, with a median time of 7.0 h to the second CT, a time point at which the majority of contusions have generally stopped expanding,^1^ it is unlikely that scan timing altered our results in a significant way. Third, all patients who underwent their first CT scan within 12 h of injury were included, which is a larger time window than the 3 h used in the CRASH-3 trial ^25^ and could have reduced the amount of CE. However, when the analysis was performed in those from Center 1 who underwent their first CT within 3 h of injury (time-from-injury data were unavailable in Center 2), absolute CE still outperformed relative CE in predicting unfavorable GOS (AUC: 0.66 vs. 0.61, *p* < 0.001) and all-cause mortality (AUC: 0.67 vs. 0.61, *p* < 0.001). In addition to this, the wide IQR of 6–12 months post-injury for assessing GOS might introduce variability, given the prolonged recovery trajectory often observed after a TBI. Outcomes for patients evaluated closer to the 6-month mark may differ from those assessed later, potentially skewing the results. Lastly, pseudo-*R^2^* values of 0.068 and 0.032 for absolute and relative CE indicate low model fit, highlighting that TBI outcomes rely on more than just CE. Still, our study's merits lie in its multi-center design, large cohort, and volumetric contusion volume calculations. Though not wholly generalizable to the TBI population, our cohort largely reflects potential future hemostatic TBI trial participants and should influence their design.

### Implementation of study results

Pending independent validation of our findings, we suggest three primary considerations for studies evaluating CE in TBI.

1. In studies focusing on lesion progression in TBI, it may be advantageous to use absolute CE as the primary outcome metric instead of relative CE.
2. Continuous absolute CE is preferable to dichotomized definitions, given the relative consistency of Youden's index across a wide range of cutoffs. If dichotomization is necessary, cutoffs between 1 and 10 mL are suitable depending on the desired sensitivity-specificity balance.
3. If studies require a baseline contusion volume ≥1 mL for inclusion, relative CE can be used, though this will reduce the patient pool and restrict generalizability.

## Conclusion

CE's correlation with patient outcomes makes it a sensible end-point for hemostatic therapy trials that are underpowered for patient-centered outcomes. Our research suggests that absolute CE is a more effective TBI outcome predictor than relative CE, especially in patients with baseline contusion volumes <1 mL. For dichotomized outcomes, cutoffs from 1 to 10 mL are appropriate, depending on the desired balance between sensitivity and specificity.

## Transparency, Rigor, and Reproducibility Summary

Because of its retrospective nature, the study was not pre-registered. The analysis plan was not formally pre-registered. The sample size was derived based on the number of patients treated at the participating hospitals during the time frame. The key inclusion criteria are established standards in the field. A total of 1869 patients were screened, out of whom 798 were included. Volume measurements were performed by investigators who were aware of relevant characteristics of the participants. All data sets were analyzed at the same time. The data sets used and/or analyzed during the current study are available from the corresponding author on reasonable request. The authors agree to provide the full content of the manuscript on request by contacting the corresponding author.

## Supplementary Material

Supplemental data

Supplemental data

Supplemental data

## Data Availability

The data sets used and/or analyzed during the current study are available from the corresponding author upon reasonable request.
