# Supplementary material for: Absolute Contusion Expansion Is Superior to Relative Expansion in Predicting Traumatic Brain Injury Outcomes: A Multi-Center Observational Cohort Study
Source: J Neurotrauma. 2024 Feb 27;41(5-6):705–13. doi: 10.1089/neu.2023.0274 (PMC10902499; doi:10.1089/neu.2023.0274)
Supplement: Supplemental data [file Suppl_TableS1.docx]

**Supplementary table 1: Antithrombotic agents and management**

| **Antithrombotic agent and management** | **Centre 1** | **Centre 2** |
| --- | --- | --- |
| **Antiplatelets** |  |  |
| Aspirin | 25 | 21 |
| Withdrawal only | 18 (72%) | 20 (95%) |
| Platelet transfusion | 7 (28%) | 1 (4.7%) |
| Clopidogrel | 3 | 1 |
| Withdrawal only | 1 (33%) | 1 (100%) |
| Platelet transfusion | 2 (67%) | 0 (0%) |
| Dual antiplatelet therapy | 4 | 4 |
| Withdrawal only | 2 (50%) | 1 (25%) |
| Platelet transfusion | 2 (50%) | 3 (75%) |
| **Anticoagulants** |  |  |
| Warfarin | 10 | 13 |
| PCC + Vitamin K | 10 (100%) | 13 (100%) |
| Apixaban | 4 | 1 |
| PCC + TXA | 4 (100%) | 1 (100%) |
| Rivaroxaban | - | 1 |
| Withdrawal only | - | 1 (100%) |
| Dabigatran | 1 | - |
| Praxbind | 1 (100%) | - |
| LMWH | - | 1 |
| Withdrawal only | - | 1 (100%) |
| **Combination** |  |  |
| Warfarin and aspirin | - | 1 |
| PCC + Vitamin K | - | 1 (100%) |
| Clopidogrel and LMWH | - | 1 |
| Withdrawal only | - | 1 (100%) |

Abbreviations: LMWH = low-molecular-weight heparin; PCC = prothrombin complex concentrate; TXA = tranexamic acid
