# Supplementary material for: Absolute Contusion Expansion Is Superior to Relative Expansion in Predicting Traumatic Brain Injury Outcomes: A Multi-Center Observational Cohort Study
Source: J Neurotrauma. 2024 Feb 27;41(5-6):705–13. doi: 10.1089/neu.2023.0274 (PMC10902499; doi:10.1089/neu.2023.0274)
Supplement: Supplemental data [file Suppl_TableS3.docx]

**Supplementary table 3: Relationship between relative contusion expansion and unfavorable Glasgow Outcome Scale**

| **Variable** | **Univariable analysis** | | | **Multivariable analysis** | |
| --- | --- | --- | --- | --- | --- |
|  | **OR (95% CI)** | **p-value** | **Pseudo-R^2^** | **OR (95% CI)** | **p-value** |
| Relative CE (%) | 1.01 (1.00–1.02) | **0.003** | 0.025 | 1.01 (1.00 – 1.02) | **0.008** |
| Baseline contusion volume (ml) | 1.01 (1.00–1.03) | **0.034** | 0.008 | 1.01 (0.99 – 1.02) | 0.479 |
| Age (years) | 1.03 (1.03–1.04) | **<0.001** | 0.123 | 1.04 (1.03 – 1.05) | **<0.001** |
| GCS on admission | 0.82 (0.78–0.86) | **<0.001** | 0.109 | 0.81 (0.76 – 0.86) | **<0.001** |
| Unilateral non-responsive pupil | 2.89 (1.79–4.79) | **<0.001** | 0.032 | 2.79 (1.51 – 5.28) | **0.001** |
| Bilateral non-responsive pupil | 4.36 (2.41–8.38) | **<0.001** | 0.042 | 3.22 (1.53 – 7.11) | **0.003** |
| Marshall CT class | 1.21 (1.10–1.33) | **<0.001** | 0.025 | 1.11 (0.98 – 1.26) | 0.089 |
| tSAH | 1.59 (1.10–2.31) | **0.014** | 0.010 | 1.39 (0.85 – 2.28) | 0.195 |
| EDH | 0.29 (0.18–0.45) | **<0.001** | 0.054 | 0.30 (0.17 – 0.52) | **<0.001** |

Abbreviations: CE = contusion expansion; CI = confidence interval; CT = computed tomography; EDH = epidural hemorrhage; GCS = Glasgow Coma Scale; OR = odds ratio; SAH = subarachnoid hemorrhage. Bold text in the p-value column indicates a statistically significant correlation (p < 0.05).
